# Performance patterns of primary health care in the face of COVID-19 in Brazil: characteristics and contrasts

**DOI:** 10.1590/0102-311XEN009123

**Published:** 2023-09-15

**Authors:** Simone Schenkman, Aylene Emilia Moraes Bousquat, Luiz Augusto Facchini, Célia Regina Rodrigues Gil, Lígia Giovanella

**Affiliations:** 1 Faculdade de Saúde Pública, Universidade de São Paulo, São Paulo, Brasil.; 2 Faculdade de Medicina, Universidade Federal de Pelotas, Pelotas, Brasil.; 3 Centro de Ciências da Saúde, Universidade Estadual de Londrina, Londrina, Brasil.; 4 Escola Nacional de Saúde Pública Sergio Arouca, Fundação Oswaldo Cruz, Rio de Janeiro, Brasil.

**Keywords:** Primary Health Care, Family Health Strategy, COVID-19, Public Health Surveillance, Health Services, Atenção Primária à Saúde, Estratégia Saúde da Família, COVID-19, Vigilância em Saúde, Serviços de Saúde, Atención Primaria de Salud, Estrategia de Salud Familiar, COVID-19, Vigilancia en Salud, Servicios de Salud

## Abstract

The adequate fight against pandemics requires effective coordination between primary health care (PHC) and health surveillance, guaranteed attention to acute and chronic demands, and a bond with the community dimension in the scope of basic health units (UBS, acronym in Portuguese). This study aims to contrast two extreme standards of PHC performance in the fight against COVID-19 in Brazil, comparing them with the profiles of the corresponding municipalities and characteristics of the organization of services. Based on the results of a cross-sectional national survey with a representative sample of UBSs, we created a synthetic index to evaluate how PHC performs against COVID-19 called CPI, composed of axes of health surveillance and social support (collective dimension) and of COVID-19 care and continuity of care (individual dimension). Of the 907 surveyed UBSs, 120 were selected, half of which had the highest indexes (complete standard) and the other half, the lowest ones (restricted standard). The municipalities of the UBSs with a complete standard are predominantly rural, have low Municipal Health Development Index (MHDI), high Family Health Strategy (FHS) coverage, and stand out in the collective dimension, whereas the UBSs in urban municipalities with this same standard have high MHDI, low FHS coverage, and an emphasis on the individual dimension. In the restricted standard, we highlight community health workers’ reduced work in the territory. In the Brazilian Northeast, UBSs with complete standard predominate, whereas, in its Southeast, UBSs with restricted standard predominate. The study poses questions that refer to the role and organization of PHC in the health care network under situations that require prompt response to health issues and indicates the greater potential capacity of the FHS program in such situations.

## Abstract (pt)

O enfrentamento adequado de pandemias requer forte articulação entre atenção primária à saúde (APS) e vigilância em saúde, atenção garantida às demandas agudas e crônicas e vinculação com a dimensão comunitária no âmbito das unidades básicas de saúde (UBS). O objetivo deste artigo é contrastar dois padrões extremos de desempenho da APS no enfrentamento da COVID-19 no Brasil, cotejando-os com os perfis dos respectivos municípios e características da organização dos serviços. A partir dos resultados de inquérito nacional transversal com amostra representativa das UBS, foi criado um índice sintético de desempenho da APS em relação à COVID-19, denominado CPI, composto pelos eixos de vigilância e apoio social (dimensão coletiva) e de atendimento ao paciente com COVID-19 e continuidade do cuidado (dimensão individual). Das 907 UBS pesquisadas, foram selecionadas 120, sendo a metade com os maiores índices encontrados (padrão completo) e a outra com os menores (padrão restrito). Os municípios das UBS com padrão completo são preponderantemente rurais, com baixo Índice de Desenvolvimento Humano Municipal (IDHM), alta cobertura da Estratégia Saúde da Família (ESF) e destacam-se na dimensão coletiva, enquanto as UBS nesse mesmo padrão situadas em municípios urbanos apresentam alto IDHM, baixa cobertura de ESF, com ênfase na dimensão individual. No padrão restrito, destaca-se a reduzida atuação de agentes comunitários de saúde no território. Na Região Nordeste, predominam UBS com padrão completo, enquanto na Sudeste preponderam UBS com padrão restrito. O estudo apresenta questões que remetem ao papel e à organização da APS na rede de cuidados em situações que requerem pronta resposta aos agravos de saúde e indica maior capacidade potencial da ESF em tais situações.

## Abstract (es)

El enfrentamiento adecuado de las pandemias requiere una fuerte articulación entre atención primaria de salud (APS) y la vigilancia en salud, una atención garantizada a las demandas agudas y crónicas y la vinculación con la dimensión comunitaria en el ámbito de las unidades básicas de salud (UBS). El objetivo de este artículo es contrastar dos patrones extremos de desempeño de la APS en el enfrentamiento del COVID-19 en Brasil, comparándolos con los perfiles de los respectivos municipios y características de la organización de los servicios. A partir de los resultados de una encuesta nacional transversal con una muestra representativa de las UBS fue creado un índice sintético de desempeño de la APS frente al COVID-19, denominado CPI, compuesto por los ejes de vigilancia y apoyo social (dimensión colectiva) y de atención al COVID-19 y continuidad de la atención (dimensión individual). De las 907 UBS investigadas, se seleccionaron 120, siendo la mitad con los índices más grandes encontrados (estándar completo) y la otra con los más bajos (estándar estricto). Los municipios de las UBS con estándar completo son preponderantemente rurales, con bajo índice de desarrollo humano municipal (IDHM), alta cobertura de la Estrategia Salud de la Familia (ESF) y se destacan en la dimensión colectiva, mientras que las UBS en este mismo estándar situadas en municipios urbanos presentan alto IDHM, baja cobertura de ESF, con énfasis en la dimensión individual. En el estándar estricto, se destaca la reducida actuación de los agentes comunitarios de salud en el territorio. En la región Nordeste predominan las UBS con estándar completo, mientras que en el Sureste predominan las UBS con un estándar estricto. El estudio aporta cuestiones que remiten al papel y organización de la APS en la red de atención en situaciones que requieren respuesta rápida a los problemas de salud e indica una mayor capacidad potencial de la ESF en tales situaciones.

## Introduction

Health emergencies, especially those on a global scale, are highly demanding on health systems and consume a large proportion of organizational resources, especially financial ones ^1^. Moreover, they disfigure and impair health care services, affecting the quality of the care provided to the population. Mitigating this situation requires a clear definition of the role of the service network of health care systems, including primary health care (PHC). PHC is central, especially in the care for vulnerable populations, ensuring greater equity. Their performance becomes even more successful when PHC, in addition to providing clinical care for acute and chronic conditions, implements health surveillance, collective action, and social support activities in its territory ^2^ ^,^ ^3^.

In Brazil, the Family Health Strategy (FHS) program, whose coverage reaches more than 130 million people, is internationally recognized, as it is the most successful model in the PHC of the Brazilian Unified National Health System (SUS, acronym in Portuguese) ^4^ and coordinates the individual (health care for users) and collective dimensions (health surveillance and social support) in its operating logic. Note that the FHS program expanded the population’s access to the health care system ^5^ ^,^ ^6^ ^,^ ^7^ and positively impacted the population’s health, such as by reducing infant and maternal mortality and deaths by preventable causes and offering hospitalizations due to conditions sensitive to the PHC ^8^ ^,^ ^9^ ^,^ ^10^ ^,^ ^11^. Thus, we can claim that the Brazilian PHC network would theoretically combine important elements to successfully cope with the COVID-19 pandemic.

Unfortunately, its success for almost three decades has been insufficient to prevent the reduction of financial, political-organizational, and personnel investments that has been carried out by neoliberal governments since 2016. In the pandemic, the potential of the FHS program was weakened by the lack of a national coordination of sanitary measures, which affected the management of the SUS in states and municipalities, contributing to relegate FHS to a subordinate role, such as actions to increase hospital and intensive care units (ICU) beds, which should have been planned together and integrated into PHC services. Moreover, PHC and health surveillance actions also proved very fragmented ^12^.

Community health workers (CHWs), who represent the strongest point of connection between PHC and health surveillance, also suffered limitations in their activities in during the pandemic due to their bond with families in the territory. Especially in 2021 ^13^, the lack of coordination in the national response, personal protective equipment (PPE), and professional training to ensure the safety of workers in community actions, restricted their presence in the territory, incisively affecting the potential performance of PHC in facing the pandemic ^14^. In the scope of the FHS program, federal management wasted CHWs’ valuable role in SUS by failing to coordinate the work of almost 300,000 workers in the health education of the population to monitor COVID-19 ^15^. Nevertheless, during the pandemic, the presence of CHWs in households and their surroundings took place by remote contact with families, favored by smartphones, tablets, and computers, indicating an exceptional capacity to carry out health surveillance ^15^. The work of tracking cases/contacts in the community, monitoring the symptoms of disease severity, providing guidelines on hygiene and prevention (including the isolation of suspected and confirmed cases) was part of the daily routine of several CHWs.

The expansion of telephone and WhatsApp contact with users ensured that the community could be monitored remotely, ensuring professionals and the population’s safety according to the local health status ^16^. In addition to its prompt adaptation in responding to the pandemic, the PHC depends on the continuity of care for users with chronic noncommunicable diseases (NCD) to ensure their great performance. Keeping users with chronic conditions at the center of care, considering their vulnerabilities to acute and infectious diseases, requires adjustments in in-person health care strategies, which also benefit from remote health care ^17^. The guarantee of access to different levels of health care refers to the comprehensiveness of the system and presupposes defined and organized flows and a corresponding scale between health care units, territory, and the population ^18^.

Brazil shows marked demographic, socioeconomic, cultural, environmental and sanitary contrasts between its five macroregions, as well as in terms of the model and infrastructure of health care services ^19^. Its South and Southeast, which have higher Human Development Index (HDI), gross domestic product (GDP) per capita, and health care infrastructure, used a strategy to face the pandemic that focused more on hospitals and a greater testing capacity ^20^, which would theoretically facilitate planning and coordinating the surveillance, isolation, and control of cases and contacts. However, this model was unable to control the pandemic as community actions were impaired and the unrestricted circulation of goods and people favored contagion and deaths, worsened by denialism and national uncoordination ^21^. Similarly, vaccination alone, despite its high coverage, was also unable to contain the pandemic given the need for joint and cohesive containment strategies in society and between SUS managers in different federative instances.

The Brazilian North and Northeast, on the other hand, despite their worse socio-sanitary condition, enjoyed a higher coverage of the FHS program, the only service available in several places. Teams face greater difficulties in referring patients to the other levels of the system. However, its potential to work with the community has managed to overcome many structural and distance-related difficulties, which not only offer barriers to access, but also protect against the arrival and transmission of COVID-19 ^22^.

A national survey, called *Primary Health Care Challenges in Fighting the COVID-19 Pandemic in the SUS* ^23^, was conducted in 2021 to evaluate the differences between ways of facing the pandemic in Brazil, based on which we selected the subject of this study. We aim to contrast two extreme standards of PHC performance in facing COVID-19, comparing them with the profiles of the corresponding municipalities and characteristics of the organization of services.

## Method

### Study design and setting

This study is part of the cross-sectional national survey conducted by the Research Network in Primary Health Care (Brazilian Association of Public Health - Abrasco, acronym in Portuguese) ^23^ called *Primary Health Care Challenges in Fighting the COVID-19 Pandemic in the SUS*, which aimed to trace the main challenges and strategies in reorganizing PHC used by basic health units (UBS, acronym in Portuguese) in coping with COVID-19.

A probabilistic sample of UBSs, registered in the Brazilian National Register of Health Establishments (CNES, acronym in Portuguese) was selected in December 2020. The sample was stratified according to the five Brazilian macroregions and its size was defined according to the number of UBSs registered in each region. The sample for the country was set to 750 units, which we expanded to 945 UBSs, considering a possible 20% loss. A 3.92 sampling error and a 1.20 design effect were obtained due to the adopted weighting.

The four PHC axes - care for patient with COVID-19, health surveillance, social support, and continuity of care - proposed by Medina et al. ^2^, composed our theoretical framework, guiding the preparation of our questionnaire and our result analysis . Data were collected out between July and November 2021. In each randomly selected UBS, a healthcare provider with complete tertiary education was invited to answer our online questionnaire. Data were collected and managed by the Research Electronic Data Capture tool (REDCap; https://redcapbrasil.com.br/), a secure web-based software platform designed to support data capture for research studies. A total of 907 responses were obtained.

The following information was collected: UBS physical structure and available connectivity resources; basic inputs and process of reorganization of care for users with COVID-19; continuity of care actions; use of telemedicine service; characteristics of access to the secondary/tertiary network (intensive clinical care); and health surveillance and social support actions in the territory.

The different draw probabilities used in the strata to choose sample units were offset by the introduction of weights in our data analysis, corresponding to the inverse of the sampling fractions used in the strata.

### Construction of the PHC performance index and its axes

A synthetic PHC performance index, called CPI (COVID PHC Index), was developed in order to express the model of organization of UBSs to cope with COVID-19. The construction of the CPI began by the definition of the relevant issues in each PHC axis ^2^. At the end of the process, 26 variables were selected, resulting in 59 questions distributed in four axes. To ensure coherence and consistency to the index, nonparametric correlations (Spearman) between the index, axes, and variables were tested, followed by factor analysis (principal component analysis) to validate its architecture, and, finally, consistency analysis (Cronbach’s alpha), resulting in the final model ^24^.

The CPI was built with equal weights for its axes and variables, according to the formula below:

$$CPI={\underset{\_}{X}}_{e_{1}}^{e_{n}}$$

where:

$$e_{*}={\underset{\overset{-}{}}{X}}_{v_{1}}^{v_{n}}$$

Axes: e_1_ to e_n_0 ≤ e_*_ ≤ 1

Variables: v_1_ to v_n_0 ≤ v_*_ ≤ 1

The CPI ranges between 100 (representing the most complete UBS performance) and 0 (indicating the nonperformance of any of the actions considered relevant in fighting COVID-19). The same scale was used to estimate the score of each axis.

Factor analysis showed that the axes that encompass collective actions (health surveillance and social support) behaved in a unified way, as opposed to the axes focused on individual actions (care for patient with COVID-19 and continuity of care). Thus, we defined two dimensions that articulate the axes and express the characteristics of the Brazilian PHC: the individual and collective dimensions.

### Definition of UBS groups with contrasting performance against the pandemic

From the total number of respondents (n = 907), 120 UBSs were selected and divided into two groups of 60, one with the highest CPI values (thus expressing a more complete standard of response to the pandemic) and the other with the lowest values (indicating a more restricted performance against COVID-19), hereinafter referred to as complete and restricted standards, respectively. This choice to analyze the two poles emerged after analyzing the complete sample, according to the index categorized by its median (which showed no marked differences). Thus, we sought to contrast the standards more clearly, with an adequate *n*, without losing statistical power.

The association of both UBS groups was analyzed according to the standard of response to the pandemic with the effect variables not included in the construction of the index: CHWs’ actions, vaccination against COVID-19, information and communications technology (ICT) infrastructure, human resources, and effects on work processes. These variables constitute structural characteristics or express the organization model of the UBSs. We also examined the association with variables extracted from secondary databases in the public domain, such as socioeconomic, demographic, political and structural variables, in addition to those of health surveillance (municipal rate of cases and mortality by COVID-19).

The variables related to the organization model and sociodemographic variables, when categorized, were compared between the two performance standards according to their proportions by Pearson’s chi-squared test. FHS coverage and local COVID-19 mortality rate were categorized according to their median in the complete sample and tested by Pearson’s chi-square. Socioeconomic, demographic, political, structural, and health surveillance variables were analyzed according to their median and interquartile range by the Mann-Whitney test, according to the index categorized in its two performance standards.

Finally, the strength of association between the variables was tested alone (bivariate analysis) and together (multivariate analysis) by odds ratio (OR), p-values, and 95% confidence intervals (95%CI), considering complex samples. In the complete, multivariate initial model, all statistically significant variables in the bivariate analysis were tested. After successive adjustments and reductions, the set of relevant variables was determined and maintained in the final model. The following parameters were considered: alpha (5%); power (80%); differences in proportions according to CHWs’ work in the territory (70% vs. 40%); and ratio between groups (1), which indicated the need for a minimum samples of 49 UBSs per group, according to the Fleiss formula adjusted for continuity ^25^.

## Results

UBSs in the restricted standard group showed significantly lower CPI medians than the complete standard group both in the collective (health surveillance and social support) and in individual dimensions (COVID-19 care and continuity of care) (Table 1).

**Table 1:** Variables related to socioeconomic, demographic, political, structural aspects, surveillance and individual and collective dimensions (CPI): distributions according to median and interquartile range (IQR) and associations with the two standards of basic health units (UBS) response to the COVID-19 pandemic. Municipalities of surveyed UBSs.

Variables	UBSs with restricted response to the pandemic [38 (18-43)]	UBSs with complete response to the pandemic [89 (85-98)]	p-value *
	Median (IQR)	Median (IQR)	
Individual dimensions (CPI)			
Care for patient with COVID-19	39 (31-50)	93 (90-95)	< 0.0001 **
Continuity of care	44 (37-55)	84 (76-90)	< 0.0001 **
Collective dimensions (CPI)			
Health surveillance	30 (20-42)	100 (90-100)	< 0.0001 **
Social support	31 (17-42)	92 (83-100)	< 0.0001 **
Socioeconomic, demographic and political variables			
MHDI ***	75 (72-78)	66 (60-62)	< 0.0001 **
GDP per capita (BRL) ^#^	36,278 (22,460-46,677)	18,399 (11,665-27,400)	< 0.0001 **
Estimated population ^#^	121,426 (53,082-373,820)	14,600 (5,376-37,884)	< 0.0001 **
% of votes in jair Bolsonaro ^##^	66 (57-77)	49 (28-61)	< 0.0001 **
Hospitals and equipment ^###^			
Hospitals (n)	4 (1.5-10)	1 (0-1)	< 0.0001 **
Respirators (n)	47.5 (12-237)	0.5 (0-6)	< 0.0001 **
ICU beds ^###^			
ICU beds ii adults - COVID-19 (n)	11 (0-35)	0 (0-0)	< 0.0001 **
ICU beds ii pediatric - COVID-19 (n)	0 (0-0)	0 (0-0)	0.6106
Total (n)	26 (10-85)	0 (0-0)	< 0.0001 **
Coverage			
FHS ^§^ (%)	63 (38-88)	100 (89-100)	< 0.0001 **
Primary care ^§^ (%)	75 (58-100)	100 (96-100)	< 0.0001 **
COVID-19 - surveillance			
Confirmed cases by municipality/100,000 ^§§^	12,251 (8.,89-14,779)	8,550 (6,123-13,117)	0.0003 **
Municipal mortality rate ^§§^	0.025 (0.019-0.033)	0.026 (0.017-0.036)	0.7648

FHS: Family Health Strategy; ICU: intensive care unit; GDP: gross dometic product; MHDI: Municipal Human Development Index.

* Mann-Whitney test;

** Significant results, p < 0.05;

*** According to the United Nations Development Program ^51^;

^#^ According to the Brazilian Institute of Geography and Statistics ^52^;

^##^ According to the Brazilian Supreme Court ^53^;

^###^ According to data from the Brazilian National Register of Health Establishments ^54^ ^,^ ^55^;

^§^ According to data from the e-Gestor AB platform ^56^;

^§§^ According to data from the Civil Registry Transparency Portal ^57^.

### Relation between the individual and collective dimensions of health care

**Figure 1:**
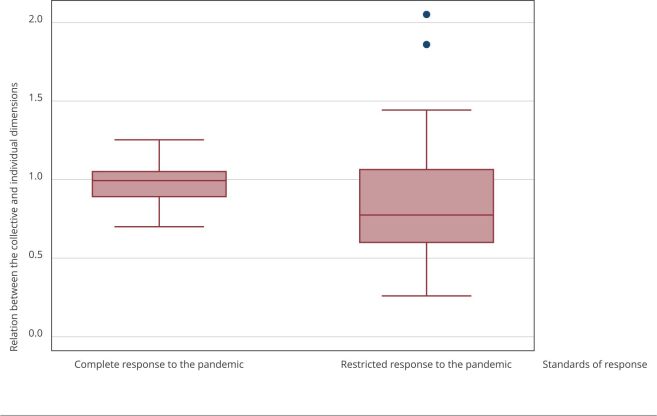
Relation between the collective and individual dimensions, according to comparison between the two standards of basic health units (UBS) response to the COVID-19 pandemic.

Complete standard UBSs showed a broader set of responses in coping with the pandemic and a more balanced relation between the individual and collective dimensions of health care, with less variability and dispersion (Figure 1). In case of a mismatch between dimensions, we observed that collective action stood out in the set of complete standard UBSs compared to individual actions and that restricted standard UBSs showed the opposite.

Restricted standard UBSs were predominantly located in municipalities with more than 100,000 inhabitants with higher Municipal HDI (MHDI), GDP per capita, and proportion of votes for Jair Bolsonaro in 2018 presidential elections (p < 0.0001). In contrast, complete standard UBSs significantly predominated in municipalities with less than 15,000 inhabitants that were poorer and had a lower proportion of votes for the president elected in 2018. The restricted standard group was also associated, at the municipal level, with a better hospital infrastructure and greater number of hospitals, respirators, and ICU beds for adults (including COVID-19 beds). In contrast, complete standard UBSs were associated with municipalities with universal coverage (100%) of the FHS program. The rate of confirmed cases per 100,000 inhabitants was significantly lower in municipalities with complete standard UBSs, with no statistical difference in municipal mortality rates (Table 1).

We found relevant associations between FHS coverage and COVID-19 mortality rates at the municipal level when we categorized medians. High FHS coverage showed a significant association (p = 0.002) with lower COVID-19 mortality rates (2-4 times), regardless of performance standard.

Complete standard UBSs predominated in the Northeast (33%), in municipalities classified by the Brazilian Institute of Geography and Statistics (IBGE) as adjacent rural (45%) and remote (12%). They had at least one FHS team (100%); computer with camera and microphone (58%); CHWs (70%); home visit (97%) and active search (88-95%); application of vaccine against COVID-19 in the UBS (88%); increased demand (95%); psychological support for healthcare providers (73%) and long COVID care (93%). They also show lower impact on the care of users with NCD (17%) (Table 2).

**Table 2:** Comparison between the two standards of basic health units (UBS) response to the COVID-19 pandemic in the reduced sample and in the complete sample, according to the results of the research and characteristics related to sociodemography, structure, surveillance and work of community health workers (CHWs) and the impact of the pandemic on primary health care (PHC), 2021.

Variables	UBSs with restricted response to the pandemic	UBSs with complete response to the pandemic	p-value *
	n (%)	n (%)	
Region			0.030 **
North	6 (10)	8 (13)	
Northeast	8 (13)	20 (33)	
Southeast	23 (38)	13 (22)	
South	16 (27)	9 (15)	
Central-West	7 (12)	10 (17)	
Rural-urban division			< 0.001 **
Adjacent intermediate	3 (5)	7 (12)	
Remote intermediate	1 (2)	0 (0)	
Adjacent rural	3 (5)	27 (45)	
Remote rural	0 (0)	7 (12)	
Urban	53 (88)	19 (32)	
FHS teams (n)			< 0.001 **
None	17 (28)	0 (0)	
1	27 (45)	37 (62)	
2-6	16 (27)	21 (35)	
More than 6	0 (0)	2 (3)	
ICT structure			
Existence of Internet	59 (98)	58 (97)	0.559
Electronic health record	47 (78)	48 (80)	0.822
Computer with camera and microphone	8 (13)	35 (58)	< 0.001 **
Activities of CHWs			
CHWs in the territory	24 (40)	42 (70)	0.001 **
CHW care for patients with respiratory symptoms	7 (12)	33 (55)	< 0.001 **
Home visit by CHWs	15 (25)	58 (97)	< 0.001 **
Active search by CHWs	10 (17)	53 (88)	< 0.001 **
Active search by CHWs for vaccination of priority groups	26 (43)	57 (95)	< 0.001 **
Active search by CHWs for those not vaccinated with 2nd dose against COVID-19	20 (33)	56 (93)	< 0.001 **
Vaccination against COVID-19 at UBS	23 (38)	53 (88)	< 0.001 **
Human resources			
Increased demand	49 (82)	57 (95)	0.023 **
Overload for health professionals	51 (85)	53 (88)	0.591
Psychological support for health professionals	26 (43)	44 (73)	0.001 **
Human resources turnover	24 (40)	23 (38)	0.852
Increased due to the pandemic	13 (22)	14 (23)	0.827
Increase - change of municipal management	9 (15)	9 (15)	1.000
Care for patients with COVID-19 sequelae	41 (68)	56 (93)	0.001 **
NCD (impact on care)			0.048 **
Improved	2 (3)	10 (17)	
Maintained	6 (10)	6 (10)	
Impaired	27 (45)	29 (48)	
Very impaired	25 (42)	15 (25)	

FHS: Family Health Strategy; ICT: information and communications technology; NCD: noncommunicable diseases.

Source: prepared by the authors.

* Chi-square test;

* p < 0.05.

Restricted standard UBSs predominated in the Southeast (38%) and urban municipalities (88%). They had at most one FHS team (73%); no CHW in the territory (60%); CHW home visit (75%); active search in the territory (57-83%); vaccine for COVID-19 in the UBS (62%); psychological support for professionals (57%) and long COVID care (32%) (Table 2).

From the point of view of the structure of UBSs, we found that four times as much complete standard UBSs (58% vs. 13%) had computers equipped with a camera and microphone (fundamental resources for telemedicine service), when compared to those with restricted standard. Regarding CHWs’ work (fundamental in the pandemic), differences between the groups were significant, with a higher frequency (from 2 to 5 times) of activities carried out in UBSs with complete standard, such as active search for patients in general and for vaccination against COVID-19, home visits, and presence of CHWs in the territory. Moreover, we observed a greater use of CHWs in care for patients with respiratory symptoms, along with the maintenance of their essential activities in the territory (Table 2).

In the complete standard group, the proportion of UBSs that performed vaccination against COVID-19 in the service itself and that reported improvement in NCD care totaled 2.3 and 5.7 times in relation to that of the restricted standard group, respectively. The negative effect of discontinuing care for NCD patients occurred in most UBSs, and 42% of the restricted standard UBSs reported great impairment to this care, compared to 25% in complete standard UBSs (Table 2).

Our bivariate logistic regression analysis showed that - compared to complete standard UBSs - restricted standard UBSs were significantly 2.7 times more likely to belong to the Southeast region and 6.9 times more likely to lack a computer with camera/microphone; 3.2 times, of CHWs working in the territory; 57.7 times, of home visits by CHWs; 39.4 times, of active search by CHWs; 19.4 times, of active search by CHWs for vaccination of priority groups; 28.2 times, of CHWs actively searching for those not vaccinated with the 2nd dose against COVID-19; 13.4 times, of vaccination against COVID-19 in the service itself; and 4.5 times, of psychological support for healthcare providers (Table 3).

**Table 3:** Comparative bivariate and multivariate analysis of the two standards of basic health units (UBS) response to the COVID-19 pandemic and variables related to sociodemography, structure, surveillance and impact of the pandemic on primary health care (PHC).

Performance standard with restricted response	OR	95%CI	p-value
Bivariate analysis			
North Region	0.80	0.25-2.58	0.703
Northeast Region	0.28	0.11-0.73	0.009 *
Southeast Region	2.70	1.17-5.26	0.002 *
South Region	2.14	0.84-5.46	0.111
Central-west Region	0.74	0.25-2.17	0.578
Lack of Internet	0.60	0.05-6.75	0.673
Lack of electronic health record	1.27	0.49-3.29	0.617
Lack of computer with camera and microphone	6.90	2.62-18.19	< 0.001 *
CHWs are not predominantly in the territory	3.22	1.43-7.26	0.005 *
CHW care for patients w/ respiratory symptoms	0.10	0.04-0.28	< 0.001 *
Lack of home visit by CHWs	57.74	12.06-276.45	< 0.001*
Lack of active search by CHWs	39.38	12.77-121.40	< 0.001*
Lack of active search by CHWs, for vaccination of priority groups	19.39	5.17-72.70	< 0.0001 *
Lack of active search by CHWs, for vaccination of those not vaccinated with the 2nd dose against COVID-19	28.23	8.03-99.21	< 0.001 *
Lack of vaccination against COVID-19 at UBS	13.36	4.75-37.60	< 0.001 *
FHS teams (n)	0.85	0.73-0.98	0.021 *
Increased demand	0.20	0.04-0.88	0.033 *
Overload for health professionals	0.89	0.28-2.82	0.840
Lack of psychological support for health professionals	4.54	2.07-9.99	< 0.001 *
Human resources turnover	1.14	0.51-2.54	0.741
Increase due to the pandemic	0.96	0.38-2.41	0.924
Increase - change of municipal management	1.00	0.34-2.93	0.999
Care for patients with COVID-19 sequelae	0.19	0.06-0.62	0.006 *
NCD (positive impact)	0.19	0.05-0.77	0.002 *
Final model			
Lack of home visit by CHWs	70.08	2.65-1,851.31	0.011 *
Lack of active search by CHWs	12.30	1.90-79.55	0.009 *
Lack of vaccination against COVID-19 at UBS	14.65	2.82-76.21	0.002 *
Lack of psychological support for health professionals	17.51	1.56-196.29	0.021 *
Care for patients with COVID-19 sequelae	0.03	0.01-0.20	< 0.001 *
Constant	0.16	0.04-0.71	0.016 *

95%CI: 95% confidence interval; CHW: community health worker; FHS: Family Health Strategy; NCD: noncommunicable diseases; OR: *odds ratio*.

Note: adjusted final model: F = 6.08; p = 0.0001; n = 120.

* p < 0.05.

**Figure 2:**
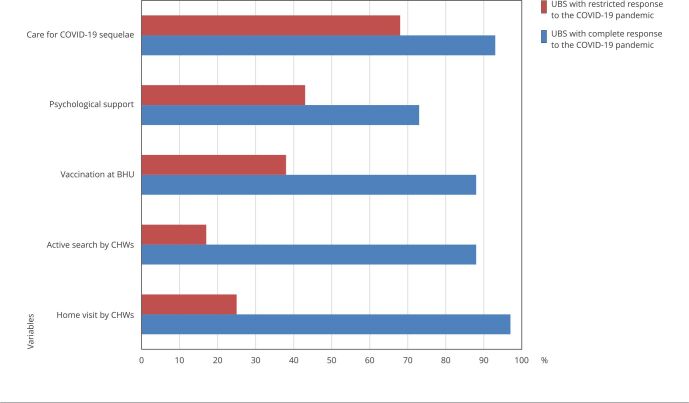
Comparison between the two standards of basic health units (UBS) response to the COVID-19 pandemic, of the proportions of variables remaining in the final logistic regression model.

Table 3 shows our final adjusted logistic regression model between the two groups of UBSs, in the fight against the pandemic. The model kept variables related to CHWs’ home visits and active search, vaccination at the UBS, psychological support for healthcare providers, and care for patients with COVID-19 sequelae. Adjusted OR showed a significant increase in the chance of lacking home visits by CHWs and psychological support, compared to crude OR. Figure 2 shows the proportions of these variables between the two groups of UBSs, with very marked differences, especially for variables regarding the work of CHWs in the territory.

CHW: community health worker.

## Discussion

The option of contrasting extremes is important as it depicts the main tensions in the Brazilian PHC setting at this crucial moment in the health context, providing elements for the discussion of SUS organization models. Moreover, contrasts enable us to understand more clearly some of the many factors that contributed to more restricted responses of PHC, a situation that was also found in other realities ^26^.

Undoubtedly, the COVID-19 pandemic was faced differently in PHC when considering Brazilian regions as the scale of analysis, shedding light on the strengths and weaknesses of the adopted models, with clearer outlines of structural and socio-environmental issues ^23^. Note that UBSs in the complete standard group predominated in rural locations, with lower MHDI and GDP per capita but with full FHS coverage and CHWs working in the territory, highlighting actions in the collective dimension. In contrast, UBSs in the restricted standard group were more present in urban locations, with high MHDI, high GDP per capita, medium or low FHS coverage, highlighting actions in the individual dimension. These disparities are related to regional social inequalities and forms of adaptation to the existing reality ^26^.

The presence of complete standard UBSs in small and rural localities is one of the highlights of this study as the UBS is often the only service available in the municipality, especially in remote rural areas ^27^. We can explain this performance by the fact that the COVID-19 pandemic required a more comprehensive response from these UBSs, organizing actions in the individual and collective dimensions ^2^ ^,^ ^28^, even if they had less access to tests and hospital infrastructure. Other studies have found the great resilience of PHC services in rural areas of South Africa ^29^ and Australia ^30^ at the beginning of the pandemic.

In the restricted standard group, most UBSs showed higher results in the individual dimension than in the collective one. In urban municipalities (with higher MHDI and per capita GDP but lower FHS coverage), performance was better for the care of patients with COVID-19, whereas rural municipalities (with high FHS coverage but lower MHDI and per capita GDP) had better performance for continuity of care. Note that ensuring continuity of care was an important challenge (often not overcome) in the most diverse contexts of the fight against the pandemic ^31^ ^,^ ^32^.

The evidence highlights the success of the FHS program to meet the health care needs of the Brazilian population, with actions to promote health, prevent risks, control diseases, and rehabilitate the population, provided by multiprofessional teams responsible for defined territories ^4^. This PHC model has a significant association with the reduction in infant and maternal mortality rates and in deaths by preventable causes ^4^. In our study, FHS coverage was associated with lower municipal COVID-19 mortality rates, regardless of the CPI. This finding reinforces the pro-equity feature of the FHS observed in several studies, as the coverage of this PHC model is higher precisely in the smallest rural localities with worse infrastructure and less access to goods and services ^33^.

Castro et al. ^34^ and Kerr et al. ^35^ point to the difficulties faced in the Brazilian North and Northeast during the pandemic, which intensified existing vulnerabilities and caused a large number of deaths among young people. Despite the difficulties and significant losses in life expectancy, these regions more actively mobilized their community components, with greater social support and measures to contain the pandemic. The Northeast showed high rates of social isolation in the first wave of the pandemic and coordination between state governments by a consortium to share best practices and innovations, with the implementation of health surveillance measures ^34^ ^,^ ^35^.

The most concerning situation is found in UBSs in areas with low FHS coverage and MHDI, hindering the fight against the pandemic and other crises. The deterioration of the Brazilian National Primary Care Policy (PNAB) since 2017 has significantly affected these localities, with reduced investment in the FHS, and the mandatory presence of CHWs in UBSs. With the implementation of the PHC reform by the Previne Brasil Program ^36^, the Expanded Family Health and Basic Healthcare Center (NASF-AB) service was discontinued, showing a low commitment to prevention. Thus, two fundamental pillars (community participation and comprehensiveness) are threatened, especially in localities with structural needs ^37^ ^,^ ^38^.

The strong association between better CPI performance and higher FHS coverage indicates a more comprehensive response to the four evaluated components, emphasizing coordinated surveillance actions in the collective dimension ^39^ ^,^ ^40^.

A considerable portion of restricted standard UBSs lacked FHS teams (28%), which is an additional problem, especially in contexts of greater inequality. The lack of FHS teams and CHW actions in the territory ^41^ could increase the chances of high mortality rates ^8^ since precisely the neediest populations neither benefit from home visits, active search nor from telemedicine service, relevant mechanisms to prevent complications and deaths ^42^.

Another key issue is the capacity to provide institutional telemedicine service beyond the pandemic. Even complete standard UBSs lack equipped computers, and their availability in UBSs in general is even lower (28%) ^43^. This result shows the recent discontinuity of political-institutional investments in telemedicine, such as the Brazilian National Telehealth Program Network, created in 2007, whose objective was precisely to strengthen the FHS and PHC, providing UBSs with greater technical and resolutive capacity ^44^ ^,^ ^45^. Note the need for investments in organizational innovation, ICTs and work optimization in PHC with priority for the FHS, considering the continental size of Brazil ^46^ ^,^ ^47^.

Psychological support for healthcare providers became a radical need during the pandemic, having a relevant impact after it ^48^. However, its lack is significant, especially in restricted standard UBSs, and more than half of them failed to guarantee it to their team. This is an important differential of complete standard UBSs in the fight against the pandemic, as well as other successful experiences found in the literature, which sought to introduce integrative practices to protect the mental health of health providers ^49^.

As for the post-pandemic impacts, the situation is far from good, even for complete standard UBSs, as most report a decrease in consultations for users with NCD, in addition to impaired care, compounded by care for post-COVID-19 sequelae. PHC professionals will play a key role in the detection and follow-up of long COVID, whose condition shows mild symptoms and usually affects patients with NCD, with an estimated prevalence of 10% to 35% of patients affected by COVID-19 ^50^.

Finally, we should highlight the importance of the lessons learned in coping with this pandemic so that managers, healthcare providers, the scientific community, and civil society conduct in-depth reflections on the hits and misses on the measures and strategies used from the perspective of social control. We also highlight the relevance of the SUS in coordinating actions and defining organizational models that effectively meet the health needs of the population, especially in the indelible role of PHC for the population of health care territories. Such measures will certainly be essential for possible future situations.

### Limitations

The reduced size of our UBS sample in the two performance standards of the CPI led to valid but little accurate results. Still, we found marked differences between groups (ignored by our analysis of the total sample).

Moreover, the associations between structural variables and outcomes were clearer and more accurate for the impact of PHC actions. Regarding health impacts, our proposal was only to approach the sets of variables and seek hypotheses since we can’t presume a causal relation. Municipal mortality rates were not associated with the index but contributed to our understanding of the protection provided by FHS coverage.

## Final considerations

The clear association between the complete standard of UBSs and FHS coverage in a municipality reaffirms the positive results of this PHC model, reinforcing the need to resume its priority in the national health policy. It is essential to maintain and expand the FHS program because of its potential to improve life quality and health in its outreach area. If, on the one hand, UBSs in municipalities with high FHS coverage managed to reduce obstacles and overcome structural difficulties, after the pandemic, they will certainly need strong coordination with the other levels of health care, especially in specialized care support strategies and necessary referrals (reduced during the pandemic).

Furthermore, the most vulnerable groups deserve special attention after the pandemic as structural and social inequalities are likely to spread over a long period, leading to varied health problems which services will need to give their attention, thus constituting an additional demand relevant to PHC.

The variables associated with performance standards in this study, such as the institutional mechanisms of telemedicine, psychological support for healthcare providers, technological innovation, and maintenance/expansion of actions in the territory, are also relevant after the pandemic, especially those carried out by CHWs. They will undoubtedly contribute with other measures to anticipate risks and expedite timely local responses according to the population’s health needs.
